# Development and Psychometric Validation of HIPER-Q to Assess Knowledge of Hypertensive Patients in Cardiac Rehabilitation

**DOI:** 10.5935/abc.20170183

**Published:** 2018-01

**Authors:** Rafaella Zulianello dos Santos, Christiani Decker Batista Bonin, Eliara Ten Caten Martins, Moacir Pereira Junior, Gabriela Lima de Melo Ghisi, Kassia Rosangela Paz de Macedo, Magnus Benetti

**Affiliations:** 1 Centro da Ciência da Saúde e do Esporte - Universidade do Estado de Santa Catarina (UDESC), Florianópolis, SC - Brazil; 2 Instituto de Cardiologia de Santa Catarina, São José, SC - Brazil; 3 Toronto Rehabilitation Institute - University Health Network, Toronto - Canada

**Keywords:** Hypertension / prevention & control, Rehabilitation, Health Education, Validation Studies as Topic

## Abstract

**Background:**

The absence of instruments capable of measuring the level of knowledge of hypertensive patients in cardiac rehabilitation programs about their disease reflects the lack of specific recommendations for these patients.

**Objective:**

To develop and validate a questionnaire to evaluate the knowledge of hypertensive patients in cardiac rehabilitation programs about their disease.

**Methods:**

A total of 184 hypertensive patients (mean age 60.5 ± 10 years, 66.8% men) were evaluated. Reproducibility was assessed by calculation of the intraclass correlation coefficient using the test-retest method. Internal consistency was assessed by the Cronbach’s alpha and the construct validity by the exploratory factorial analysis.

**Results:**

The final version of the instrument had 17 questions organized in areas considered important for patient education. The instrument proposed showed a clarity index of 8.7 (0.25). The intraclass correlation coefficient was 0.804 and the Cronbach’s correlation coefficient was 0.648. Factor analysis revealed five factors associated with knowledge areas. Regarding the criterion validity, patients with higher education level and higher family income showed greater knowledge about hypertension.

**Conclusion:**

The instrument has a satisfactory clarity index and adequate validity, and can be used to evaluate the knowledge of hypertensive participants in cardiac rehabilitation programs.

## Introduction

Cardiovascular diseases are the leading cause of mortality in the world, as a consequence of population aging and disease-related epidemiological changes,^1^ imposing high costs to health.^2^ Among these conditions, systemic arterial hypertension (SAH) stands out as a multifactorial clinical condition associated to functional, structural and metabolic changes, with consequent increase in the risk of fatal and nonfatal cardiovascular events.^3^

SAH is a serious public health problem, affecting nearly one billion people.^4^ In an important study,^5^ SAH emerges as the main risk factor in the world, and is associated with 9.4 million global deaths a year.^5^ In Brazil, the prevalence of SAH is estimated to be from 22 to 42% of adult population.^6^

Cardiac rehabilitation (CR) is one of the recommended treatments for cardiovascular diseases, consisting of a multidisciplinary approach for secondary prevention,^7^ that reduces the recurrence of cardiovascular events and mortality.^8^ The benefits of CR are mostly due to habit changes and, in this regard, patient education has been considered one of the most important approaches.^9-12^

In this context, an efficient SAH management depends on patient’s understanding about his condition and treatment.^13^ Therefore, patients that participate in education programs are more able to successfully control over their own health care. Thus, hypertensive patient’s knowledge about his condition is part of the therapeutic success, who becomes co-responsible for the treatment.^9,14,15^

Nevertheless, there are few validated tools able to provide accurate information about education of hypertensive patients. While some instruments does not focus CR,^16-19^ others include only questions deemed as relevant by the authors, without undergoing a psychometric validation.^13-15,20-23^

This gap in the knowledge opens the possibility of investigation, since assessment tools are important instruments in educational programs. These instruments enable the identification of patients’^9^ educational needs and of specific conditions involving paradigms of health and disease, which are likely to change.^10^ Thus, the aim of this study was to develop and psychometrically validate an instrument to assess the knowledge about the disease of patients enrolled in CR programs (HIPER-Q).

## Methods

### Conception and procedures

This study was approved by the research ethics committee of Santa Catarina State University (UDESC) (approval number 159.213/2012). The study had a cross-sectional, observational design.

In the first stage of the study, a literature review was performed to identify the pieces of knowledge about SAH considered relevant to hypertensive individuals.^3,24^ The bibliographic search was performed in Pubmed database from January 2010 to September 2016.

The questionnaire was constructed and revised by a commission composed of 17 health specialists, with experience in CR. These specialists carried out an analysis of content and clarity of the instruments, to verify its adequacy to hypertensive patients participating in CR programs.

The second stage was a pilot study to evaluate both applicability and reproducibility of the instrument, as well as patients’ understanding of the items (clarity). A convenience sample of hypertensive patients, who participated in CR programs, was studied, and the results were used for refinement of the HIPER-Q instrument. Patients of the pilot study did not participate in the psychometric validation.

The third step was the psychometric validation. The refined tool was used in a larger convenience sample, composed of hypertensive patients participating in CR programs at the Clinic of Cardiology and Cardiopulmonary and Metabolic Rehabilitation (Cardiosport), the Center of Cardiology and Sports Medicine (*Núcleo de Cardiologia e Medicina do Esporte,* NCME) of the clinic, and the Santa Catarina Institute of Cardiology (ICSC). Data were collected between November 2015 and May 2016.

### Participants

Patients of the pilot study and patients of the psychometric validation group were recruited from the CR programs mentioned above if they met the following inclusion criteria: clinical diagnosis of SAH, age ≥ 18 years, participation in a CR program for a period longer than one month, and agreement to participate in the study by signature of the informed consent form, according to the CNS 466/12 resolution. Patients with cognitive dysfunctions that could make the completion of the questionnaire difficult, *i.e*., who did not demonstrate a minimal understanding of socio-demographic questions were excluded, at the investigator’s discretion.

### Measurements

To assess the clarity of the instrument, participants of the study pilot were asked to classify each item of the questionnaire in a 1 (not clear) to 10 (very clear) scale.^25^ Also, these patients answered the HIPER-Q at two different occasions with a 14-day interval for analysis of the reproducibility of the instrument. Patients who participated in the psychometric validation were characterized by sex, age, educational attainment, comorbidities, time in CR, cardiac risk factors and clinical history. These characteristics were self-reported.

### Statistical analysis

Sample calculation for the psychometric analysis was performed according to Hair & Anderson’s^26^ who recommend a minimal sample size of 10 subjects per item and/or a minimum of 100 participants. Since the questionnaire was composed of 17 items, a sample of 170 hypertensive subjects was considered sufficient.

Test-retest reproducibility of the instrument was validated in the pilot study group using the intraclass correlation coefficient (ICC). The items should meet the minimal recommended standard - ICC > 0.7.^27,28^

Psychometric properties of the new tool were assessed by analysis of internal consistency, criterion validity and factorial structure. First, internal consistency was analyzed in the psychometric validation group by Cronbach's alpha, reflecting the internal correlation between items and factors.^26^ Values greater than 0.60 are generally considered acceptable.^29^ Second, criterion validity was analyzed by relating the HIPER-Q scores to patients’ educational attainment and family income, using the Spearman correlation. Third, the dimensional structure (as well as the construct validity) was evaluated by exploratory factor analysis. A component method for factor extraction was performed, considering only those factors with characteristic values > 1.0. When necessary, items with low factor loading (< 0.35) were excluded.

Once the factors were selected, a correlation matrix was generated, in which the associations between items and factors were identified by factorial loadings greater than 0.30 in only one factor. The promax method was used for matrix interpretation,^30^ and the Spearman correlation was used for analysis of criterion validity.

Finally, a descriptive analysis of HIPER-Q was performed using mean values and standard deviations of normally distributed variables, and median and interquartile ranges for variables with non-normal distribution. Data normality was evaluated by the Kolmogorov-Smirnov test. Due to non-normality of data, we used the chi-square test to evaluate the association between the HIPER-Q scores based on patients’ sociodemographic and clinical characteristics. Patients’ total knowledge was represented by the median of total score.

Statistical analyses were performed using the *Statistical Package for Social Sciences (*SPSS) version 20 (IBM Inc. 2011, NYC), and the level of significance was set at 5% for all tests.

## Results

### Participants

For content validation, 17 health professionals with experience in CR were consulted: 6 (35.5%) physicians, 6 physiotherapists (35.3%), 2 nurses (11.8%), 2 physical educators (11.8%) and 1 dietitian (5.9%). For the pilot test, 30 hypertensive patients participating in CR programs were recruited by convenience to answer the questionnaire; 11 (22%) of them were women, with mean age of 62 ± 8 years.

For psychometric validation, 184 hypertensive patients with mean age of 60.5 ±10 years and median time of diagnosis of 8 years (interquartile range 18 years) completed the HIPER-Q. Of these patients, 101 (54.9%) were retired. Participants’ characteristics are described in Table 1.

**Table 1:** Socioeconomic and clinical characteristics of hypertensive patients (n = 184) and HIPER-Q ratings (median and interquartile range) according to these characteristics

Variable	Category	n(%)	HIPER-Q score Median (IR)	p^†^
Sex	Male	123(66.8)	25 (10)	0.033*
	Female	61(33.2)	27 (8.5)	
Comorbidities	CAD	149(81)	25 (8.5)	0.033*
	Dyslipidemias	149(81)	25 (8.5)	0.127
	Myocardial infarction	127(69)	24 (8)	0.003*
	Diabetes Mellitus	52(28.3)	25 (10)	0.493
	POAD	24(13)	27 (10)	0.805
	Stroke	23(12.5)	26 (11)	0.928
	Smoking	03(1.6)	26 (0.0)	0.998
	COPD	02(1.1)	35 (0.)	0.539
Cardiologic procedures	Angioplasty	116(63)	24 (7.5)	0.019*
	Cardiac surgery	53(28.8)	23 (8)	0.275
Classes of antihypertensive drugs	ACEI	65(35.3)	28 (10.5)	0.768
	α and β adrenergic blockers	56(30.4)	28 (10.75)	0.186
	Angiotensin II receptor antagonists	52(28.3)	25 (9)	0.669
	Diuretics	21(11.4)	27 (14)	0.820
	Calcium channel blockers	05(2.7)	29 (17)	0.195
	Unknown	15(8.2)	22 (7.75)	0.755
Number of anti-hypertensive drugs	0	37(20.1)	22 (7)	0.993
	1	108(58.7)	26 (10)	
	2	36(19.6)	28 (9.75)	
	3	2(1.1)	23 (0.0)	
Type of rehabilitation	Public	162(88)	25 (9)	0.274
	Private	22(12)	32.5 (10.25)	
Time of rehabilitation	From 01 to 06 months	105(47.3)	26 (9)	0.317
	From 06 to 12 months	10(4.5)	22 (14.25)	
	From 12 to 24 months	17(7.7)	27 (10)	
	Over 24 months	51(23)	27 (10)	
Family income (salary)	< 01	09 (4.9)	22 (8.5)	0.023*
	01 - 05	94(51.1)	25.5 (9)	
	05 - 10	42(22.8)	26 (8)	
	10 - 20	32(17.4)	31 (14.5)	
	> 20	07(3.8)	35 (11)	
Educational level	Never went to school	08(4.3)	20 (8.75)	0.002*
	Some primary education	59(32.1)	25 (7)	
	Completed primary	20(10.9)	27 (6.5)	
	Some high school	16(8.7)	22.5 (4.75)	
	Completed high school	35(19)	27 (11)	
	Some college	13(7.1)	31 (14)	
	Completed college	30(16.3)	31.5 (14.25)	
	Graduate degree	3(1.6)	36 (0.0)	

IR: interquartile range; CAD: coronary artery disease; POAD: peripheral obstructive arterial disease; COPD: chronic obstructive pulmonary disease; ACEI: angiotensin converting enzyme inhibitors

† chi-square;

* p < 0.05.

### Development of HIPER-Q

The literature review on health education for hypertensive patients in CR programs revealed consistent findings between the articles. The first version of the HIPER-Q was developed based on literature data. Nineteen items were constructed encompassing seven important domains in patient education: self-care, treatment, diagnosis, physical exercise, concept and pathophysiology, signs and symptoms and risk factors. Similar to other educational instruments,^12,31^ for each item, one answer is considered the “most correct” one and receives score 3, and another answer is considered “partially corrected” and receives score 1. The other two answer options - the incorrect option and the “don’t know” option receives no score (zero). According to the classification described in Table 2, the sum of the scores represents mean total knowledge, where the maximum score of 51 points corresponds to ‘perfect’ knowledge.

**Table 2:** Classification of patient's knowledge by HIPER-Q score

Sum of the scores	Percentage	Classification of knowledge
From 46 - 51 points	90 - 100%	Excellent
From 36 - 45 points	70 - 89%	Good
From 25 - 35 points	50 - 69%	Acceptable
From 15 - 24 points	30 - 49%	Poor
< 15 points	< 30%	Insufficient

### Clarity validation

The construction rules of the item sources and of the theoretical analysis of the items, content and semantics were considered ‘clear’ by 79% of the specialists, with a median clarity score of 8.5 (0.75). However, most of the items received comments on their semantic contexts. Each item was widely discussed by the authors, and all changes suggested by the specialists were accepted. This version of the questionnaire was analyzed by the same professionals, and the final version was then provided, with 96% of agreement between the items and median clarity score of 9.54 (0.30).

### Pilot study

The average time for completion of the questionnaire by the participants (n = 30) was 15.4 ± 2.2 minutes. The median clarity score was 8.7 (0.25), and no item had a clarity score lower than 7.0, indicating that the questionnaire was well understood by the target population.

### Test-retest reproducibility

Total ICC of the instrument was 0.804, obtained by the final test-retest scores.^27^ The items “Also with respect to systemic arterial hypertension, we can affirm that” and “What is the best diet for patients with systemic arterial hypertension?” had a ICC lower than 0.7 (0.43 and 0.58, respectively) and were excluded from the final version,^27^ which was then composed of 17 questions. The ICC of each question is presented in Table 3

**Table 3:** Score of the HIPER-Q items (n = 184) (median and interquartile range), and intra class correlation coefficient of each item (n = 30)

Domain	Questions	HIPER-Q score in median (interquartile range)	ICC
Self-care	9.If a health professional finds that your blood pressure is altered, you should	3 (0)	0.72
	15. With respect to self-measurement of blood pressure, it is correct to say that	0 (1)	0.96
	17. With respect to systemic arterial hypertension patient's self-care, it is correct to say that:	1 (3)	0.79
Treatment	6. What is the ideal treatment to reduce blood pressure levels?	1 (2)	0.75
	14. Which of these drugs aim to reduce blood pressure levels?	3 (2)	0.80
Diagnosis	5. Which among the items listed below are the most accurate in the diagnosis of systemic arterial hypertension?	1 (1)	0.82
	16. About the "white coat syndrome", it is correct to say that	0 (3)	0.85
Physical exercise	4. Physical exercise for systemic arterial hypertension patients should:	1 (3)	0.81
	8. The practice of physical exercises is contraindicated when the patient:	1 (3)	0.76
	10. On the basis of your knowledge about systemic arterial hypertension, answer the following:	3 (0)	0.81
	11. What favorable changes are systemic arterial hypertension patients able to obtain with the regular practice of physical exercises?	1 (3)	0.82
Concept and pathophysiology	1. Systemic arterial hypertension is:	1 (3)	0.80
	13. What are the main consequences of untreated systemic arterial hypertension?	3 (2)	0.76
Signs and symptoms	3. With respect to systemic arterial hypertension symptoms, check the correct answer	1 (3)	0.81
Risk factors	2. Which of the risk factor groups below has the greatest influence on the development of systemic arterial hypertension?	3 (2)	0.81
	7. Which systolic arterial pressure and diastolic arterial pressure values, respectively, are recommended for systemic arterial hypertension patients?	1 (3)	0.75
	12. With respect to stress, we can say that:	1 (3)	0.78

ICC: intra-class correlation coefficient.

### Psychometric validation

The HIPER-Q was administered to participants of CR programs, and the mean scores of the questionnaire items are shown in Table 3. Overall, the HIPER-Q showed a moderate internal consistency (Cronbach's alpha = 0.648).

With respect to criterion validity, a relationship of HIPER-Q total score was found with educational attainment and family income. Weak positive correlations were found of knowledge level with educational attainment (rho = 0.346; p < 0.01) and family income (rho = 0.176; p = 0.017).

Dimensional structure was evaluated by exploratory factor analysis. The Kaiser-Meyer-Olkin (KMO = 0.669) test and the Bartlett’s sphericity test (*X^2^* 2066.56; p < 0.001) indicated adequacy of data for factor analysis. Five factors were extracted and, together, they accounted for 51.1% of the total variance of the items, whose characteristic values were > 1.1. Table 4 displays the factor loadings of the items. Factor “1” reflects “General Conditions”, and is responsible for 18.8% of total variance, whereas the other factors had a lower influence of the variance. Factor “2” reflects “Treatment”; factor “4” reflects “Physical Exercise”; factor 4 reflects “risk factors” and factor 5 reflects “self-care’.

**Table 4:** Classification of the HIPER-Q factorial structure by loadings

Item	Domain	Factors				
		1	2	3	4	5
17	Self-care	0.825				
6	Treatment	0.792				
5	Diagnosis	0.745				
11	Physical exercise	0.664				
1	Concept and pathophysiology	0.477				
14	Treatment		0.646			
13	Concept and pathophysiology		0.631			
3	Signs and symptoms		0.525			
16	Diagnostic			0.734		
4	Physical exercise			0.63		
8	Physical exercise				0.635	
7	Risk factors				0.534	
12	Risk factors				0.470	
2	Risk factors				0.328	
10	Physical exercise					0.684
9	Self-care					0.580
15	Self-care					0.426

### Descriptive analysis

The instrument had a median total score of 26 (10). In patients’ classification, a high prevalence (44.6%) of “acceptable knowledge” was observed. Patients showed greater knowledge about the items: “If a health professional says that your blood pressure is altered, you should”, “On the basis of your knowledge about systemic arterial hypertension, answer the following:” and “Which of the risk factor groups below has the greatest influence on the development of systemic arterial hypertension?”. The lowest level of knowledge was seen for the items: “With respect to self-measurement of blood pressure, it is correct to say that”, “About the *white coat syndrome*, it is correct to say that” and “Which among the items listed below are the most accurate in the diagnosis of systemic arterial hypertension?”. Regarding the knowledge domains, patients showed higher level of knowledge in the areas - “disease” and “concept and pathophysiology”. On the other hand, the lowest level of knowledge was shown for the “diagnostic” and “signs and symptoms” domains.

As shown in Table 1, greater knowledge about SAH was associated with coronary artery disease (p < 0.001), dyslipidemias (p = 0.006), myocardial infarction (p < 0.001) and peripheral obstructive arterial disease (p = 0.004). In addition, previous angioplasty (p < 0.001) or cardiac surgery (p = 0.002) was associated with greater knowledge about the disease.

## Discussion

Patient’s education is one of the central components of CR, and is crucial for promoting the understanding about secondary prevention strategies and adherence to treatment.^9,28,31^ In the present study, a new tool for the assessment of knowledge in hypertensive patients enrolled in CR programs was developed and psychometrically validated by a rigorous process. In general, clarity, internal consistency, reliability, dimensional structure and criterion validity were established, indicating the validity and usefulness of the HIPER-Q in the assessment of hypertensive patients’ knowledge about the disease.

The first data to be considered is the clarity index, generated by professionals and patients, demonstrating that the instrument proposed can be easily understood by the study population.^31,32^ Second, comparisons of the factorial analysis reported in similar studies^12,31,33^ revealed that the HIPER-Q showed similar arrangement of factors and items; in each of the five factors, those items with similar knowledge domains were predominant in the instruments. The factors were clustered by stability, interpretation of the areas and basic principles of construction rules, in order to establish a reliable, consistent construct. In each domain, the factors included different amounts of terms that were correlated with each other, which may be explained by the fact that SAH is characterized as a systemic, multifactorial disease.^3,24^

Results of internal consistency (Cronbach’s alpha = 0.648) were consistent with those reported in previous studies involving instruments of assessment of hypertensive patients’ knowledge about their conditions,^19^,^34^-^36^ and studies with similar structure.^12,33^ This indicates an adequate correlation between the items of the questionnaire. Nevertheless, the HIPER-Q was validated in public and private CR programs with different characteristics, which may have affected the alpha value (not as high as those of similar studies).

Regarding the criterion validity, both educational attainment and family income were related to the knowledge about SAH. These findings suggest that socioeconomical factors are determinants of knowledge about health, as previously demonstrated.^12,22,31,33^

The current study also evaluated the level of knowledge of the sample patients, who showed an overall knowledge classified as “acceptable”. Our findings, supported by other authors,^13,18-21^ reflect the importance of evaluating the knowledge about health and formulating hypothesis that elucidate the determining factors of the information gaps. Therefore, patient education is an important component of CR programs^9,28^ and is associated with a successful self-management of disease and patient’s behavior changes.^33^

We did not find in the literature, longitudinal studies demonstrating the effects of a higher level of knowledge about SAH on outcomes, such as worse prognosis or mortality. Thus, one may expect that the HIPER-Q can be used in this regard in future studies. In this context, studies on other chronic diseases have shown promising results, suggesting that disease-related education may be determinant in the control of risk factors, such as sedentary lifestyle, smoking and continuity of treatment, which may lead to reductions in comorbidities, health costs and even mortality.^34,35^

In this scenario, there is a lack of instruments to measure the knowledge about the disease in participants of CR.^31^ Most of the studies reviewed have only developed SAH questions deemed as relevant by the authors,^13,14,20-23^ without conducting a psychometric validation as performed in the present study.^25,36^ In addition, other validated studies have not specifically evaluated the knowledge of hypertensive patients in CR.^16-19,37-39^ Therefore, our study aimed to develop an instrument to healthcare professionals, capable of establishing educational strategies directed to patients’ needs,^12,31^ and that would help in the evaluation and planning of the educational process of hypertensive subjects in CR programs.

Caution is needed in interpreting these findings. First, the results cannot be generalized, due to the facts that the sample was selected by convenience, and only three CR programs were included, which affects the achievement of the outcomes. Second, the development of the instrument proposed was based on consensus and guidelines, which encompass numerous SAH-related issues not necessarily covered by CR programs. Third, although all patients included were participants of CR programs, the programs were different (of public and private nature), with different approaches, which may have influenced the results. Fourth, the instrument was not developed using plain language techniques, or “simple” language, which may have created difficulties in the interpretation of the items, and consequently affected the results.^36^ Fifth, the current study did not achieve the sample size recommended by the test-retest procedure.^36^ Sixth, participants were not asked about their occupations, which may also have influenced the results, since patients graduated in medicine and/or other health-related areas, for example, may have had greater chance of giving correct answers. Further studies are needed to evaluate whether the HIPER-Q is sensitive to longitudinal changes by assessing patients’ knowledge before and after their participation in CR programs.

## Conclusion

The present study demonstrated that the HIPER-Q showed sufficient reliability, consistency and validity, corroborating its use in future studies to evaluate the knowledge of SAH patients in CR programs. This instrument is expected to support the assessment of the educational component of CR programs and to identify the knowledge that is compatible with patients’ need for information.

## References

[1] Roth GA, Forouzanfar MH, Moran AE, Barber R, Nguyen G, Feigin VL (2015). Demographic and epidemiologic drivers of global cardiovascular
mortality. N Engl J Med.

[2] Myers L, Mendis S (2014). Cardiovascular disease research output in WHO priority areas
between 2002 and 2011. J Epidemiol Glob Health.

[3] Malachias MV, Souza WK, Plavnik FL, Rodrigues CI, Brandão AA, Neves MF, Sociedade Brasileira de Cardiologia (2016). 7ª Diretriz Brasileira de hipertensão
arterial. Arq Bras Cardiol.

[4] Ibrahim MM, Damasceno A (2012). Hypertension in developing countries. Lancet.

[5] Lim SS, Vos T, Flaxman AD, Danaei G, Shibuya K, Adair-Rohani H (2012). A comparative risk assessment of burden of disease and injury
attributable to 67 risk factors and risk factor clusters in 21 regions,
1990-2010: a systematic analysis for the Global Burden of Disease Study
2010. Lancet.

[6] Brasil, Ministério da Saúde (2015). Vigitel Brasil 2014- Vigilância de fatores de risco e
proteção para doenças crônicas por
inquérito telefônico.

[7] Herdy AH, López-Jiménez F, Terzic CP, Milani M, Stein R, Carvalho T (2014). South American guidelines for cardiovascular disease prevention
and rehabilitation. Arq Bras Cardiol.

[8] Anderson L, Oldridge N, Thompson DR, Zwisler AD, Rees K, Martin N (2016). Exercise-based cardiac rehabilitation for coronary heart
disease. Cochrane Database Syst Rev.

[9] Kayaniyil S, Ardern CI, Winstanley J, Parsons C, Brister S, Oh P (2009). Degree and correlates of cardiac knowledge and awareness among
cardiac impatiens. Patient Educ Couns.

[10] Hacihasanoglu R, Gözüm S, Capik C (2012). Validity of the Turkish version of the medication adherence
self-efficacy scale-short form in hypertensive patients. Anadolu Kardiyol Derg.

[11] Buckley JP, Furze G, Doherty P, Speck L, Connolly S, Hinton S (2013). BACPR scientific statement: British standards and core components
for cardiovascular disease prevention and rehabilitation. Heart.

[12] Bonin CD, dos Santos RZ, Ghisi GL, Vieira AM, Amboni R, Benetti M (2014). Construction and validation of a questionnaire about heart
failure patients' knowledge of their disease. Arq Bras Cardiol.

[13] Dawes MG, Kaczorowski J, Swanson G, Hickey J, Karwalajtys T (2010). The effect of a patient education booklet and BP 'tracker' on
knowledge about hypertension A randomized controlled trial. Fam Pract.

[14] Strelec MA, Pierin AM, Mion Jr D (2003). The influence of patient's consciousness regarding high blood
pressure and patient's attitude in face of disease controlling medicine
intake. Arq Bras Cardiol.

[15] Almas A, Godil SS, Lalani S, Samani ZA, Khan AH (2012). Good knowledge about hypertension is linked to better control of
hypertension; a multicenter cross sectional study in Karachi,
Pakistan. BMC Res Notes.

[16] Batalla Martínez C, Blanquer Laguarta A, Ciurana Misol R, Garcia Soldevilla M, Jordi Cases E, Pérez Callejón A (1984). Cumplimiento de la prescrición farmacológica em
pacientes hipertensos. Aten primaria.

[17] Martins D, Gor D, Teklehaimanot S, Norris K (2001). High blood pressure knowledge in an urban African-American
community. Ethn Dis.

[18] Leblanc ME, Cloutier L, Veiga EV (2011). Knowledge and practice outcomes after home blood pressure
measurement education programs. Blood Press Monit,.

[19] Peters RM, Templin TN (2008). Measuring blood pressure knowledge and self-care behaviors of
African Americans. Res Nurs Health.

[20] Familoni BO, Ogun SA, Aina AO (2004). Knowledge and awareness of hypertension among patients with
systemic hypertension. J Natl Med Assoc.

[21] Vieira AJ, Cohen LW, Mitchell CM, Sloane PD (2008). High blood pressure knowledge among primary care patients with
known hypertension: a North Carolina Family Medicine Research Network
(NC-FM-RN) study. J Am Board Fam Med.

[22] Sanne S, Muntner P, Kawasaki L, Hyre A, DeSalvo KB (2008). Hypertension knowledge among patients from an urban
clinic. Ethn Dis.

[23] Adams OP, Carter AO (2011). Knowledge, attitudes, practices, and barriers reported by
patients receiving diabetes and hypertension primary health care in
Barbados: a focus group study. BMC Fam Pract.

[24] James PA, Oparil S, Carter BL, Cushman WC, Dennison-Himmelfarb C, Handler J (2014). 2014 evidence-based guideline for the management of high blood
pressure in adults: report from the panel members appointed to the Eighth
Joint National Committee (JNC 8). JAMA.

[25] Pasquali L (2003). Psicometria: teoria dos testes na psicologia e na
educação.

[26] Hair JF, Anderson RE (1998). Multivariate data analysis.

[27] Dancey CP, Reidy J (2005). Statistics without maths for Psychology: using SPSS for Windows.

[28] Ghisi GL, Grace SL, Thomas S, Evans MF, Oh P (2013). Development and psychometric validation of a scale to assess
information needs in cardiac rehabilitation: The INCR Tool. Patient Educ Couns.

[29] Nunnally JP (1978). Psychometric theory.

[30] Kaiser HF (1960). The application of eletronic computers to fator
analysis. Educ Psycol Meas.

[31] Ghisi GL, Durieux A, Manfroi WC, Herdy AH, Andrade A, Benetti M (2010). Construction and validation of the CADE-Q for patient education
in cardiac rehabilitation programs. Arq Bras Cardiol.

[32] Martins GA (2006). Sobre confiabilidade e validade. Revista Brasileira de Gestão e Negócios (RBGN).

[33] Ghisi GL, Grace SL, Thomas S, Evans MF, Oh P (2015). Development and psychometric validation of the second version of
the Coronary Artery Disease Education Questionnaire (CADE-Q
II). Patient Educ Couns.

[34] Schwarzer R, Lippke S, Luszczynska A (2011). Mechanisms of health behavior change in persons with chronic
illness or disability: the health action process approach
(HAPA). Rehabil Psychol.

[35] Ghisi GL, Abdallah F, Grace SL, Thomas S, Oh P (2014). A systematic review of patient education in cardiac patients do
they increase knowledge and promote health behavior change?. Patient Educ Couns.

[36] Terwee CB, Bot SD, de Boer MV, van der Windt DA, Knol DL, Dekker J (2007). Quality criteria were proposed for measurement properties of
health status questionnaires. J Clin Epidemiol.

[37] Erkoc SB, Isikli B, Metintas S, Kalyoncu C (2012). Hypertension Knowledge-Level Scale (HK-LS): a study on
development, validity and reliability. Int J Environ Res Public Health.

[38] Chatziefstratiou AA, Giakoumidakis K, Fotos NV, Baltopoulos G, Brokalaki-Pananoudaki H (2015). Translation and validation of the Greek version of the
hypertension knowledge-level scale. J Clin Nurs.

[39] Kim MT, Song HJ, Han HR, Song Y, Nam S, Nguyen TH (2012). Development and validation of the high blood pressure-focused
health literacy scale. Patient Educ Couns.

